# Impact of gas emboli and hyperbaric treatment on respiratory function of loggerhead sea turtles (*Caretta caretta)*

**DOI:** 10.1093/conphys/cox074

**Published:** 2018-01-12

**Authors:** Cyril Portugues, Jose Luis Crespo-Picazo, Daniel García-Párraga, Jordi Altimiras, Teresa Lorenzo, Alicia Borque-Espinosa, Andreas Fahlman

**Affiliations:** 1 Fundación Oceanogràfic de la Comunidad Valenciana, Gran Vía Marqués del Turia 19, 46005Valencia, Spain; 2 AVIAN Behavioral Genomics and Physiology, Department of Physics, Chemistry and Biology, Linköping University, Linköping 581 83, Sweden; 3 Marine Biology Laboratory, Zoology Department, University of Valencia. Doctor Moliner n° 50, 46100 Valencia, Spain; 4 Grupo de Investigación Biomédica en Imagen GIBI230, Radiology Department, Hospital Universitario y Politécnico La Fe, Av. Bulevard Sur, 46026 Valencia, Spain

**Keywords:** Diving physiology, decompression sickness, lung function, comparative physiology

## Abstract

Fisheries interactions are the most serious threats for sea turtle populations. Despite the existence of some rescue centres providing post-traumatic care and rehabilitation, adequate treatment is hampered by the lack of understanding of the problems incurred while turtles remain entrapped in fishing gears. Recently it was shown that bycaught loggerhead sea turtles (*Caretta caretta*) could experience formation of gas emboli (GE) and develop decompression sickness (DCS) after trawl and gillnet interaction. This condition could be reversed by hyperbaric O_2_ treatment (HBOT). The goal of this study was to assess how GE alters respiratory function in bycaught turtles before recompression therapy and measure the improvement after this treatment. Specifically, we assessed the effect of DCS on breath duration, expiratory and inspiratory flow and tidal volume (*V*_T_), and the effectiveness of HBOT to improve these parameters. HBOT significantly increased respiratory flows by 32–45% while *V*_T_ increased by 33–35% immediately after HBOT. Repeated lung function testing indicated a temporal increase in both respiratory flow and *V*_T_ for all bycaught turtles, but the changes were smaller than those seen immediately following HBOT. The current study suggests that respiratory function is significantly compromised in bycaught turtles with GE and that HBOT effectively restores lung function. Lung function testing may provide a novel means to help diagnose the presence of GE, be used to assess treatment efficacy, and contribute to sea turtle conservation efforts.

## Teaser

We investigated lung function in bycaught sea turtles with and without gas emboli. Hyperbaric O_2_ treatment significantly improves lung function. Pulmonary function testing may be a useful diagnostic tool to assess fisheries induced trauma.

## Introduction

Decompression sickness (DCS), or ‘the bends’, is caused by the formation of gas bubbles, most commonly N_2_, in blood and tissues (also called gas embolism) after reduction in pressure occurs during ascent from a dive (Mahon and Regis, 2014). In air breathing vertebrates, DCS symptoms range in severity from skin rashes and tissue distortion to central nervous system disorders, paralysis and respiratory difficulties that commonly reverse during recompression or hyperbaric O_2_ treatment (HBOT) (Vann *et al.*, 2011; Mahon and Regis, 2014). Recompression therapy is based on the assumption that the higher pressure will compress the gas bubbles and force the gas back into solution, which will resolve the existing gas emboli (GE). In the chamber, recompression is followed by a slow decompression, which helps to safely remove the increased tissue and blood gas burden via the lungs through respiration, while preventing bubbles from forming again. The increased partial pressure of O_2_ during HBOT improves oxygenation and helps increase the gas washout (Mahon and Regis, 2014). In a recent study, GE was reported in bycaught loggerhead sea turtles (García-Párraga *et al.*, 2014). The GE were similar to those reported in marine mammals (Bernaldo de Quirós *et al.*, 2012), and it was concluded that the GE in the turtles was clear evidence of DCS. Of 67 bycaught turtles, 29 were found to have GE, and the bubbles resolved upon recompression treatment. These results provided a definitive diagnosis that marine vertebrates can experience DCS, at least under unnatural circumstances. The authors found that turtles with a high number of bubbles often had respiratory distress and frequently died (García-Párraga *et al.*, 2014; Fahlman *et al.*, 2017a), the extent to which GE affects the respiratory system is not known.

Sea turtles possess multicameral lungs with structural features that are similar to marine mammals, including strongly reinforced large diameter airways and homogeneously distributed parenchyma containing smooth muscle and fibrous connective tissue (Tenney *et al.*, 1974; Lutcavage *et al.*, 1987, 1989). These structural features allow high respiratory flows (Tenney *et al.*, 1974). In addition, the vital capacity (VC) in marine mammals and sea turtles is almost as large as the total lung capacity (TLC). Thus, these animals can exchange almost the entire lung volume in a single breath (Berkson, 1966; Lutz and Bentley, 1985). However, unlike marine mammals, the current knowledge suggests that the turtle lungs are the major O_2_ store instead of the blood (Burggren, 1988). Loggerhead sea turtles are reported to expire when surfacing and breath before they dive on full inspiration (Lutcavage *et al.*, 1987). This breathing pattern suggests that the lung is used as an O_2_ store during diving (Lapennas and Lutz, 1982). It has been suggested that passive lung collapse, caused by alveolar compression as pressure increases, is the main mechanism by which turtles prevent N_2_ uptake while diving (Berkson, 1967). In addition to the passive pulmonary shunt, turtles have a three-chambered heart which allows blood to bypass the lungs through intra-ventricular communication (also known as a right to left shunt, R–>L), and in some species a muscular sphincter on the pulmonary artery may be vital to regulate pulmonary blood flow and to shunt blood, offering additional protection against the bends (Lutcavage and Lutz, 1991; García-Párraga *et al.*, 2014).

During intense exercise or stress, as may occur when turtles are entrapped in fishing nets, the shunt mechanism may be suppressed by the activation of the sympathetic nervous system which may increase N_2_ uptake (Fahlman *et al.*, 2009; García-Párraga *et al.*, 2014; Lorenzo Bermejo et al., 2016; Fahlman, 2017). As a consequence, bubbles may form during or after ascent and cause emboli, which reduces blood flow and further prevents gas exchange and inert gas removal (Vann *et al.*, 2011; Fahlman, 2017). In addition, excessive gas bubble formation in tissues and systemic vasculature may also cause coelomic organ distention, which compresses the lungs against the carapace (García-Párraga *et al.*, 2014). This reduces respiratory function and further reduces gas exchange and bubble elimination.

Previous clinical observations illustrated that turtles with a high GE density often showed reduced lung fields on diagnostic imaging (radiography and CT-scan) and experienced respiratory dyspnea/distress that would reverse following HBOT (García-Párraga *et al.*, 2014). Therefore, the objective of the present work was to investigate and quantify the effect of GE on lung function in loggerhead sea turtles by performing opportunistic lung function testing on bycaught individuals with different degree of GE (Fahlman *et al.*, 2017a). Lung function testing was performed before and at regular intervals after hyperbaric chamber treatment throughout the recovery period, in order to assess the level of respiratory compromise post-capture and the efficacy of HBOT in recovery of normal breathing performance.

## Material and methods

### Facility and animal acquisition

The study was carried out at a rehabilitation centre (Área de recuperación y conservación de fauna marina-ARCA) located at and managed by the Fundación Oceanogràfic in Valencia, Spain. The facility has a permit from the Valencian Regional Government to hold and rehabilitate stranded and bycaught sea turtles. The Animal Care and Welfare Committee at the facility approved all spirometry procedures (Animal care number: OCE-22-16). A total of 21 loggerhead turtles were used in this study. Most turtles were accidentally caught by fishermen of the Valencian coast of Spain and brought directly to the facility. Animals were admitted between February and November 2016 from local gillnet and trawling fisheries, or from recreational boaters that retrieved turtles floating at the sea surface. Through an on-going collaboration with the local government and the local fishermen, detailed information is requested by each boat that reports a turtle fisher interaction. These reports include for each turtle, the date of capture, cause of capture, the depth at which the commercial gear was set, sea surface temperature, capture location as well as the condition and behaviour immediately after capture (Table 1).

**Table 1:** Animal ID (ID), body mass (*M*_b_), bubble grade (GE score), fisheries interaction type/cause of arrival (cause), hyperbaric O_2_ treatment (HBOT, yes—Y or no—N), arrival and release date and curved carapace length (CCL)

ID	*M*_b_ (kg)	GE score	Cause	HBOT (Y/N)	Arrival date	Release date	CCL (cm)
T242	20.6	1	Gill net	N	17 February 2016	23 March 2016	56.0
T243^a^	22.7	1	Trawl	N	22 February 2016	16 March 2016	56.4
T246	21.6	2	Gill net	Y	4 March 2016	30 March 2016	54.2
T249	16.3	2	Trawl	Y	23 March 2016	6 April 2016	51.5
T250	6.8	2	Gill net	Y	23 March 2016	6 April 2016	38.7
T255	16.9	2	Gill net	Y	7 April 2016	27 April 2016	40.5
T245	22.0	3	Trawl	Y	4 March 2016	6 April 2016	54.5
T247	4.6	3	Trawl	Y	9 March 2016	27 April 2016	33.8
T252	7.9	3	Trawl	Y	30 March 2016	19 April 2016	40.5
T248	21.2	4	Trawl	Y	9 March 2016	19 April 2016	56.5
T244	38.5	0	Trawl	N	4 March 2016	30 March 2016	65.0
T258	3.9	0	Gill net	N	15 April 2016	15 August 2016	32.0
T259	4.2	0	Gill net	N	16 April 2016	27 April 2016	36.0
T260	5.4	0	Gill net	N	16 April 2016	27 April 2016	33.0
T262	8.7	0	Floating surface	N	24 April 2016	22 July 2016	35.8
T270	19.0	0	Gill net	N	12 July 2016	26 July 2016	54.5
T271	1.2	0	Stranded	N	18 July 2016	15 August 2016	23.0
T272^b^	1.6	0	Stranded	N	18 July 2016	11 August 2016	25.6
T273	14.0	0	Gill net	N	4 August 2016	13 October 2016	48.0
T274	6.9	0	Floating surface	N	9 August 2016	13 October 2016	39.4
T275	13.5	0	Gill net	N	5 September 2016	13 October 2016	54.5

^a^Individual with mild water aspiration.

^b^Individual diagnosed with general infection and septicaemia. Only individuals with a GE score >1 underwent HBOT, while turtles without GE or with a GE score ≤1 were placed immediately in the water tanks.

The turtles were housed individually in circular tanks, either 2 m (tank A) or 5 m (tank B) in diameter with a water depth of 0.95 m. The water temperature ranged from 18.1 to 24.9°C, with a salinity of 37 g/L and pH of 7.5. The tanks were connected to a water filtration system that continuously circulated and treated water by mechanical filtration, protein skimming, ozone, UV light and a heating-cooling system. The tanks were housed in a building with artificial light with a 12-h photoperiod (8:00–20:00).

#### Veterinary examination

Upon arrival, all turtles underwent a health examination including complete physical exam, weight, morphometric measurements, radiography, ultrasound and blood sample collection. The presence and severity of GE were determined by radiographs and ultrasound examination. The severity of GE was scored on a 5-point scale as follows: no intravascular gas detected, very mild, mild, moderate, moderate to severe, or severe as previously detailed (García-Párraga *et al.*, 2014; Fahlman *et al.*, 2017a).

### Hyperbaric oxygen treatment

Approximately 30–60 min after admission at the veterinary clinic, HBOT was performed in a custom built hyperbaric chamber (41 cm × 77 cm, internal height and diameter).

Recompression was done using pure O_2_ from a pressurized medical O_2_ cylinder. The duration inside the hyperbaric chamber varied for each individual according to GE severity, but in most cases, it was around to 12–14 h. The turtles were initially compressed to 2.6 ATA. For the next 12 h, the chamber pressure decreased progressively to 1.6–1.8 ATA, and within 2 h the pressure was back at 1 ATA.

Following chamber treatment, a full veterinary assessment was again performed, including physical exam, radiography and ultrasound to evaluate the resolution of GE. Finally, the turtle was placed in a holding tank at the rehabilitation centre under daily observation until release. Turtles were released when they were feeding, swimming and behaving normally and when the blood values were completely normalized.

### Lung function testing

Lung function testing was done using an ultrasonic spirometer (True-Flow spirometer, NDD Medical Technologies Inc. Andover MA) that measured respiratory flow. The turtles were placed in the water with the head out. The spirometer was connected to the smallest possible animal anaesthesia face-mask (Henry Schein, mask diameter 44–130 mm). The head of the turtle was placed inside the mask through a rubber gasket. The mask allowed the animal to breathe freely through the flow metre, and the rubber gasket prevented air leakage. The mask size was selected to ensure fit, and minimize dead space. The spirometer was connected to a computer and the data were obtained through custom-written software (WBreath MFC Application version 3.40.5.0). Each lung function test lasted ~10 min, with one person lightly restraining the turtle at a slight angle in the water by the fore limbs without impeding normal ventilation, while a second person kept the spirometer attached to the turtle.

The respiratory flow trials allowed us to determine breathing frequency (*f*_R_), breath duration, and tidal volume (*V*_T_, amount of air flowing in and out of the lungs during breathing at rest). Lung function testing was performed upon arrival (Day 0). Next, all turtles with a GE score >1 underwent recompression/HBOT treatment (*n* = 8, Table 1), while animals with a GE score ≤1 were placed directly in their holding tank (*n* = 13). All turtles underwent a second lung function test on Day 1; the measurement on animals with GE score >1 were performed directly following the veterinary examination after the chamber treatment, while the measurements for animals without GE and GE score ≤1 were done approximately at the same time of Day 0. Lung function testing was repeated each week until release following the same methodology with the participation of two people.

### Data processing and statistical analysis

All gas volumes were standardized to temperature pressure dry conditions (STPD, Quanjer *et al.*, 1993). Exhaled air was assumed saturated with water vapour at the estimated body temperature of the turtle. As sea turtles are ectothermic, it was assumed that the body temperature was equal to the water temperature where the turtle was held, therefore, tank temperatures were taken daily and included in the calculations. Inhaled air volume was corrected for ambient temperature, ambient pressure and relative humidity.

Respiratory data are reported as average values for all complete breaths. For evaluating the effect of HBOT on respiratory variables, we used a paired *t*-test. The temporal relationship between a respiratory variable (breath? duration, respiratory flow and *V*_T_), and number of breaths since the beginning of the measurements (bn), and/or number of days since arrival at the rehabilitation centre (days) were analysed using linear-mixed effects models (lme, R: A Language and Environment for Statistical Computing, R Foundation for Statistical Computing, version 3.1.1, 2014). The individual animal was treated as a random effect, which accounted for the correlation between repeated measurements on the same individual (Littell *et al.*, 1998). Initially, variables were selected for inclusion in a multivariate model if the univariate analysis had a *P* < 0.2 (Wald’s test). Best models were chosen by the Akaike information criterion (AIC) against nested models and significance determined using the Likelihood ratio test (LRT). Individuals with at least two spirometry measurements were considered to investigate the temporal changes during rehabilitation. In this study, *P*-values ≤ 0.05 were considered as significant and *P* ≤ 0.1 were considered a trend. Data are presented as the mean ± standard deviation (SD), unless otherwise stated.

*f*_R_ was determined as number of breaths divided by the total length of the measurement period (≈10 min). Minute ventilation (${\dot{V}}_{E}$) was calculated as the product of average expired volume *V*_T_ and breathing frequency.

## Results

Lung function testing was performed in 21 bycaught loggerhead turtles, 11 without GE (confirmed on diagnostic imaging), and 10 with GE. Table 1 summarizes morphometrics, GE score (García-Párraga *et al.*, 2014; Fahlman *et al.*, 2017a), cause of admission to the rehabilitation centre, as well as the accession and release dates.

### Effect of recompression treatment

No changes in breath durations were found 12 h after HBOT (*n* = 8, inspiratory, *T*_insp_ = 1.52 ± 0.44 s expiratory, *T*_exp_ = 1.40 ± 0.37 s; total breath duration, *T*_tot_ = 2.64 ± 0.61 s, *P* > 0.1 for all, paired *t*-test), nor did any of these variables change from Day 0 to Day 1 in animals that did not exhibit GE and undergo HBOT (*n* = 11, *T*_insp_ = 1.11 ± 0.31 s; *T*_exp_ = 1.03 ± 0.26 s; *T*_tot_ = 2.07 ± 0.38 s, *P* > 0.1 for all, paired *t*-test).

**Figure 1.**
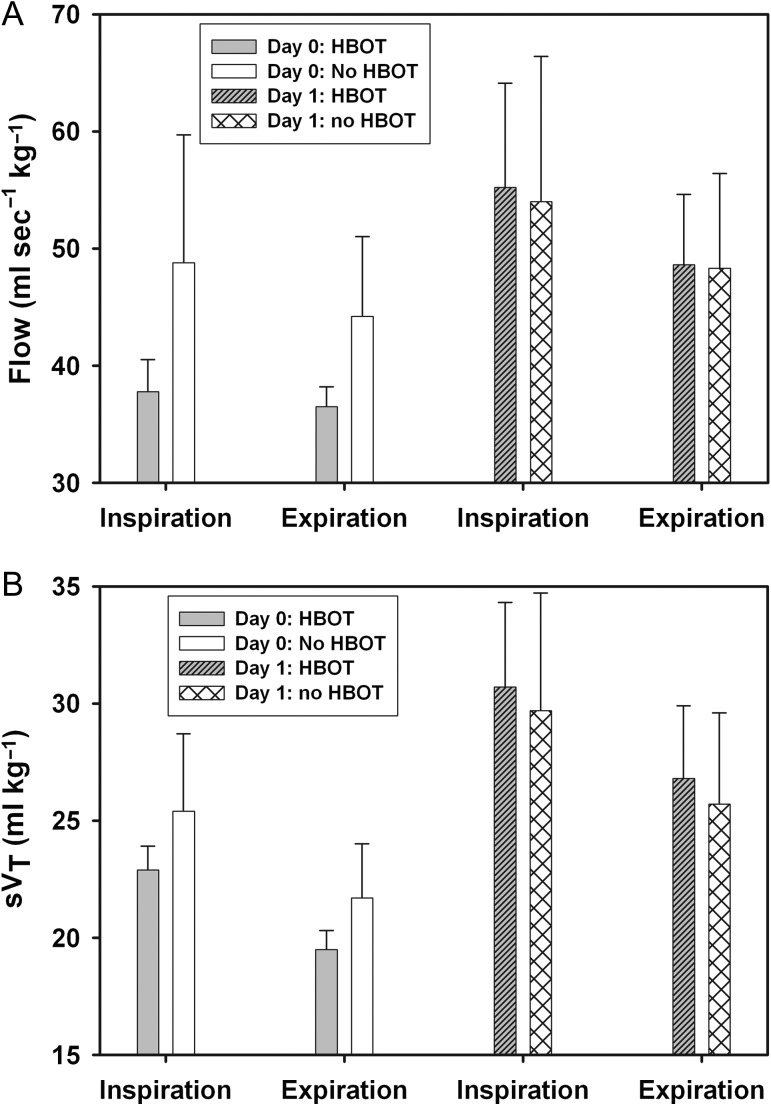
Inspiratory and expiratory (**A**) mass-specific respiratory flow (flow) and (**B**) mass-specific tidal volume (s*V*_T_) on Day 0 (arrival) and 1. Turtles with a gas emboli (GE) score >1 underwent hyperbaric O_2_ treatment (HBOT) on Day 0, while turtles without GE or with a GE score ≤1 (no HBOT) were placed in water. The following day all turtles were tested again. Both respiratory flow and s*V*_T_ significantly increased for both groups from Day 0 to Day 1 (*P* < 0.05).

In turtles that underwent HBOT (*n* = 8), the expiratory flow increased an average of 32% and the inspiratory flow increased an average of 45% following HBOT (paired *t*-test, *P* < 0.05). In animals that did not have GE and were not exposed to HBOT (*n* = 11), the respiratory flow also increased but an average of 9 and 11%, respectively, for expiratory and inspiratory flow (paired *t*-test, *P* < 0.05, Fig. 1A).

In turtles with GE score >1 that underwent HBOT (*n* = 8), both expiratory and inspiratory *V*_T_’s increased following treatment an average of 35% and 33%, respectively (paired *t*-test, *P* < 0.05). Changes in *V*_T_ were not correlated with GE score (*P* > 0.1). In turtles without GE (*n* = 11), the expiratory and inspiratory *V*_T_ increased an average of 18 and 17%, respectively (paired *t*-test, *P* < 0.05, Fig. 1B).

There were no changes in ${\dot{V}}_{E}$ from Day 0 to Day 1 for turtles with (non-parametric sign test, *P* = 0.34, *n* = 8) or without GE and GE score ≤1 (non-parametric sign test, *P* = 0.22, *n* = 11).

### Temporal changes during rehabilitation

A subset of turtles (*n* = 19) were tested throughout recovery and participated in spirometry measurements on a weekly basis. As mentioned previously, only turtles having two or more spirometry measurements were considered for the analysis.

Body mass (*M*_b_) was not important to explain inspiratory, expiratory or total breath duration (all *P* > 0.3, Wald’s test). For total (LRT: 72, *P* < 0.01, df = 3), inspiratory (LRT: 14, *P* < 0.01, df = 3) and expiratory (LRT: 142, *P* < 0.01, df = 3) breath duration the most parsimonious models included days following treatment (range: 1–96 days) and number of breaths after placing the mask over the head (bn, maximum number of breaths during a trial range: 7–142):

$$\begin{array}{c} \mathrm{Inspiratory}\;\mathrm{duration}\left(s\right)\;=0.978+0.00332\;*\;\mathrm{bn}+\mathrm{day}\;*\;0.00282\\ \;–\;0.0009793\;*\;\mathrm{bn}\;*\;\mathrm{day}\\ \mathrm{Expiratory}\;\mathrm{duration}\left(s\right)=0.960+0.00585\;*\;\mathrm{bn}+\mathrm{day}\;*\;0.00773\\ \;–\;0.000150\;*\;\mathrm{bn}\;*\;\mathrm{day}\\ \;\mathrm{Breath}\;\mathrm{duration}\left(s\right)=1.906+0.00900\;*\;\mathrm{bn}+\mathrm{day}\;*\;0.106\\ \;–\;0.000225\;*\;\mathrm{bn}\;*\;\mathrm{day} \end{array}$$

Thus, all breath durations increased slightly with time (repeated spirometry trials) and throughout a trial, but the effect decreased with time (the cross-term between bn and day).

For both expiratory (LRT: 6.2, *P* < 0.01, df = 1) and inspiratory (LRT: 5.1, *P* < 0.01, df = 1) flows, the most parsimonious model included *M*_b_:

$\begin{array}{cc} \mathrm{Expiratory flow}\;\left(L\;\cdot \;s^{-1}\right) & =0.178+0.0377\;*\;M_{b}\\ \mathrm{Inspiratory flow}\;\left(L\;\cdot \;s^{-1}\right) & =0.201+0.0300\;*\;M_{b} \end{array}$

No differences were found for inspiratory and expiratory flows for turtles with or without GE during rehabilitation. The average (±SE, *n* = 19) inspiratory and expiratory flows were respectively, 53 ± 35 and 47 ± 23 mL s^−1^ kg^−1^.

According to the model, *V*_T_ changed with *M*_b_ and the number of days following treatment.

For both expiratory (LRT: 26, *P* < 0.01, df = 2) and inspiratory *V*_T_ (LRT: 16, *P* < 0.01, df = 2), *M*_b_, and days following treatment (day) warranted inclusion in the model:

$\begin{array}{cc} \mathrm{Expiratory volume} & =0.0750+0.0215\;*\;M_{b}+0.00172\;*\;\mathrm{day}\\ \mathrm{Inspiratory volume} & =0.0368+0.0219\;*\;M_{b}+0.0239\;*\;\mathrm{day} \end{array}$

No differences were found for inspiratory and expiratory tidal volume for turtles with or without GE during rehabilitation. The average (±SE, *n* = 19) inspiratory and expiratory mass-specific *VT* were, respectively, 29 ± 14 mL s^−1^ kg^−1^ and 26 ± 11 mL s^−1^ kg^−1^.

## Discussion

We investigated respiratory function in 21 loggerhead sea turtles that were opportunistically tested after having been bycaught in local fisheries. The radiographs revealed that one animal had aspirated some water (Table 1), which could have interfered with the effect of GE when assessing lung performance. While we could not control for age, sex or certain diseases among individuals, our results indicate that GE may limit the ability to remove the elevated gas burden following the decompression insult. The treatment effect did not vary in turtles with different GE score. After treatment, there was a temporal increase in both respiratory flow and *V*_T_ in all individuals. There was a small but significant change in respiratory function in animals without GE from the day they arrived until the following day. These changes may be caused by changes in respiratory function as the turtles are kept out of water for several hours during transport to the rehabilitation centre, progressive resolution of pulmonary edema following water aspiration, or stress.

Lung function testing, or spirometry, is a minimally invasive method to assess lung health and is commonly used in humans to diagnose a variety of respiratory diseases (Miller *et al.*, 2005). Spirometry may be a useful diagnostic tool in veterinary medicine, but while it is minimally invasive it offers some challenges. For example, placement of the mask on the head may cause a certain amount of stress, and the effect of this stress may be reduced with repeated sessions as the animal becomes used to the procedure. In fact, there were small but significant changes in *V*_T_ and respiratory flow for animals with or without GE on Day 2 onwards, possibly indicating gradual lung re-expansion after being placed back into the water, pulmonary edema resolution in case of water aspiration (detectable on radiographs in one case) or a certain level of stress during initial sessions that was reduced during repeated sessions.

In the current study, the respiratory function for turtles both with or without GE were statistically significant between D0 and D1, but the increase in respiratory flows (32–45% vs. 9–11%, Fig. 1A) and *V*_T_ (35–33% vs. 18–17%, Fig. 1B) following HBOT was much greater as compared with turtles without GE. The increase in respiratory flows might be a consequence of lung re-expansion in turtles that have spent several hours on deck, or following reabsorption of the N_2_ bubbles in the blood or coelomic organs that prevented full lung re-expansion. In future studies lung function testing should be considered for turtles shortly after being brought back to the surface. Previous work has shown that turtles with moderate to severe GE that do not receive hyperbaric O_2_ treatment are likely to die (García-Párraga *et al.*, 2014). We propose that with increasing GE the tissue expansion compress the lungs against the carapace, which reduces the efficiency of ventilation and gas exchange. This is particularly exacerbated in sea turtles where the body wall has very limited distensible capacity and extrapulmonary gas distributed in coelomic organs would impede normal respiration. Hyperbaric treatment helps compress the bubbles and increases gas solubility, which helps re-dissolve the bubbles in the blood and tissues. This improves the circulation and gas exchange, both which are important to help remove the intravascular and tissue gas bubbles (Fahlman, 2017). Following HBOT, most of the gas was removed, which in turn helped to increase *V*_T_ and respiratory flow. The change in respiratory variables in turtles without GE may indicate the potential effect of stress caused by the spirometry measurement, or stabilization of turtles following transport to the rehabilitation centre. As the changes in respiratory variables from D0 to D1 were significantly lower in turtles without GE as compared with those with GE and HBOT. The continued changes in respiratory variables during rehabilitation may indicate that the turtles continued to improve or that repeated testing helped desensitize the animals. However, we conclude that HBOT had a significant effect on lung function.

Data on respiratory function in loggerhead sea turtles are scarce (Lutcavage *et al.*, 1987, 1989; Lutcavage and Lutz, 1991). Maximal mass-specific expiratory flow from excised lungs ranged from 18–25 mL s^−1^ kg^−1^ (BTPS, body mass range: 0.5–86 kg), while the highest flow in three spontaneously breathing individuals on land was 22 mL s^−1^ kg^−1^ (Table 2, Lutcavage *et al.*, 1989). The expiratory flow in the current study was considerably higher (28–124 mL s^−1^ kg^−1^, Table 2) as compared with the values reported in the previous studies. The measurements in the current study were performed in water whereas in former studies the measurements were done on land (Table 2). Previous time spent on land and body weight may have influenced the expiratory flows in the previous studies. However, in two turtles that we measured both on land and in water there was no indication that either inspiratory or expiratory flow differed significantly between the two environments. In the current study we report volumes in STPD, while BTPS is more commonly used in respiratory research. Another potential difference could be that repeated measurements help desensitize the turtles and reduce potential stress caused by the experimental procedure, which may lead to higher flows and volumes as the animals are more relaxed.

**Table 2:** Published data for average (±SD) breath duration (*T*_tot_, s), mass-specific respiratory flow (s$\dot{V}$, mL s^−1^ kg^−1^), mass-specific tidal volume (s*V*_T_, mL kg^−1^). The range of values observed are within parenthesis. The reference for each study is included in the last column for loggerhead turtles (*Caretta caretta*)

Species (number of animals)	*M*_b_ (kg)	*T*_tot_ (s)	*s*$\dot{V}$ (mL s^−1^ kg^−1^)	s*V*_T_ (mL kg^−1^)	Reference
*Caretta caretta* (5)^a,b^	7–18			33.2 ± 10.8 (21–49)	(Lutcavage and Lutz, 1991)
*Caretta caretta* (7)	0.5–86	Exp: 1.8 ± 0.59; Insp: 1.9 ± 1.1	Excised: (18–25); Spontaneous: 16 ± 8 (7222)	(23–31)	Lutcavage *et al.* (1989)
*Caretta caretta* (8)^a^	4.3–22.7			22 ± 2 (≈10–32)	Lutz *et al.* (1989)
*Caretta caretta* (10)^a^	1.2–38.5	Exp: (0.74–1.47) ± 0.28; Insp: (0.84–1.28) ± 0.17	Exp: (28.5–124.2) ± 30; Insp: (25.2 – 170) ± 45.8	Exp: (16.3–61.9) ± 14.5; Insp: (18.9–75.9) ± 18.3	Current study

^a^Measurements performed in water while in previous studies measurements were performed on land.

^b^Indicates studies where volumes were reported in BTPS. The values reported for the current study come from non-affected animals after Day 1 (Table 1). One animal in the current study (T272) was diagnosed with septicaemia, a disease altering lung function, and is not included on this table.

Mass-specific *V*_T_’s from turtles without GE are similar to the values found in previous studies for loggerhead sea turtles, ranging from 20 to 50 mL kg^−1^ (Table 2, Lutcavage *et al.*, 1989; Lutz *et al.*, 1989; Lutcavage and Lutz, 1991).

During lung function testing in humans it is common to instruct the patient to perform maximal respiratory efforts. Such cooperation is possible with humans or trained animals, e.g. maximal respiratory efforts made in dolphins (Fahlman *et al.*, 2015). In non trained individuals under spontaneous ventilation, lung function testing may have a more limited value as a diagnostic tool as these maximal efforts provide important information about flow limitations and minimize variation between breaths (Quanjer *et al.*, 1993; Miller *et al.*, 2005; Fahlman *et al.*, 2017b; Wanger *et al.*, 2005). Therefore, spirometry could be especially useful for trained animals that perform forced inhalation and exhalations. In the current study, all animals came from the wild and temporarily stayed at the rehabilitation centre before being released back into the sea, which prevented a potential training bias. Repeated measurements had a still significant but much smaller effect on respiratory variables as compared with HBOT on GE affected individuals. Thus, our data due clearly indicate the efficacy of lung function testing as a diagnostic tool to assess respiratory function in turtles with GE. While limitations when working with wild animals should be considered, there is potential for lung function testing as a minimally invasive method to assess respiratory performance in veterinary medicine. This technique may also allow lung function monitoring over time in a rehabilitation setting for instance in wild populations suffering from lung disease after being exposed to toxic chemicals (Smith *et al.*, 2012, 2017). Further research with the data obtained from this study could provide us with new insights into the effects of DCS and the mechanisms underlying the recovery. It may also provide a simple, portable and minimally invasive diagnostic tool to help assess GE presence and its impact in bycaught turtles.

The present data are important to assess the effects of fisheries interactions and how disease, treatment and time on rehabilitation alter respiratory function in these species. Hopefully, our results can be used to improve basic respiratory physiology in sea turtles and the impact of GE in respiration and gas exchange. While HBOT might help save bycaught sea turtles, a better understanding of the effect of bycatch may help mitigate fisheries interactions altogether. The results of this study are important to establish a better understanding about the physiological challenges that human-made changes to the environment may cause, and allow us to better protect these species to assure their survival.
